# Amelogenin: a Protein to Smile About

**DOI:** 10.1371/journal.pbio.1000263

**Published:** 2009-12-22

**Authors:** Caitlin Sedwick

**Affiliations:** Freelance Science Writer, San Diego, California, United States of America

**Figure pbio-1000263-g001:**
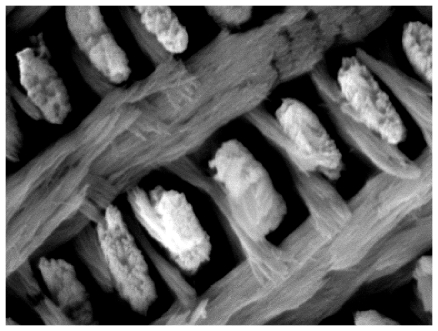
New evidence reveals how polyproline-repeat elements of enamel proteins evolve from amphibians to mammals to refine biological apatite structure and function. The striking microarchitecture of biological minerals is evident in tooth enamel, above.

[Fig pbio-1000263-g001]How did we humans (and other vertebrates) get our sparkling smiles? The hard white coating on our teeth, the enamel, is made up of hydroxyapatite—the same mineral that forms our bones. In our teeth, hydroxyapatite is organized into parallel arrays of columnar apatite crystals called prisms. The growth and organization of enamel prisms is controlled by special proteins secreted by cells known as ameloblasts. One of these proteins, amelogenin, forms tiny aggregates known as nanospheres once it has been secreted. Amelogenin nanospheres are thought to help apatite crystals form and grow.

Studies of amelogenin's amino acid sequence have revealed that short regions at either end of the protein are found throughout vertebrate evolution. In fact, one of the main differences between the frog amelogenin gene and that found in higher vertebrates (for example, mammals) is found in the protein's polyproline domain (so-called because the amino acid proline appears with high frequency in this region). This area is an “evolutionary hotspot” that is significantly longer in mammals than in lower vertebrates. In this issue of *PLoS Biology*, Tianquan Jin, Tom Diekwisch, and colleagues shed new light on the evolutionary significance of differences in the polyproline domain and on its role in the regulation of apatite crystal formation.

Amelogenin's polyproline domain contains several repeats of an amino acid triplet: a proline followed by two other amino acids (an arrangement known as a PXX repeat). When Jin and colleagues compared the frequency of PXX repeats in different vertebrates, they found that that the number of PXX repeats is higher in mammals than in lower vertebrates. For example, the longest continuous PXX stretch in frog amelogenin has only 6 PXX repeats, compared to 21 repeats in bovine amelogenin. This finding led the authors to ask whether amelogenin from different species assembles into different sized aggregates. Indeed, when they compared frog, mouse, goat, and bovine nanospheres, they found that the diameter of amelogenin nanospheres from the different species was inversely correlated with the length of the species' PXX repeats.

The authors' studies on native amelogenin proteins suggest that PXX repeat length directly influences the dimensions of assembled amelogenin nanospheres. To test this idea, the group created custom polypeptides with different PXX repeat lengths (12, 24, or 33 repeats), allowed the peptides to coassemble into nanospheres, and measured the resulting structures. Again, they found that longer PXX repeats yielded nanospheres of smaller dimensions. This finding led Jin and colleagues to explore whether PXX repeat length influences apatite crystal growth; they added the custom polypeptides to a hydroxyapatite crystallization solution and measured the lengths of the resulting crystals. The results of this experiment showed that longer PXX repeats promote the growth of thinner but longer apatite crystals.

The authors next wondered whether a similar effect could be observed in vivo. To address this question, Jin and colleagues created transgenic mice expressing the frog amelogenin gene in place of the mouse amelogenin gene. Compared to wild-type mice, the mutant mice exhibited a 50% thinner enamel layer. What's more, the mutants' enamel was poorly organized and lacked the highly organized prism structure normally found in mouse tooth enamel. In fact, the enamel of the mutant mice more strongly resembled frog tooth enamel, which lacks prisms, than it did mouse tooth enamel.

Finally, the authors investigated how increasing PXX repeat length allows for smaller nanospheres by using NMR spectroscopy to estimate the 3-D shape of their custom polypeptides. These studies showed that the shortest polypeptide is very disordered. However, the longest polypeptide tended to adopt a conformation known as a “polyproline II helix”, which is known to pack into a very compact shape. The ability of longer PXX repeats to pack more tightly, combined with the hydrophobic nature of the polyproline region and the reduced thermodynamic mobility of a longer molecule, could explain the smaller nanospheres formed by longer PXX repeat proteins, the authors say.

Taken together, Jin and colleagues' data indicate that PXX repeat length plays a strong role in shaping both amelogenin nanosphere dimensions and in organizing the resulting enamel growth. The authors theorize that because smaller amelogenin nanospheres are more tightly packed in the extracellular matrix, they would promote more efficient bundling and organization of the apatite crystals they nucleate than would larger amelogenin nanospheres. These findings could have interesting implications for our understanding of how genes controlling mineral growth were incorporated into the vertebrate evolutionary path.

**Jin T, Ito Y, Luan X, Dangaria S, Walker C, et al. (2009) Elongated Polyproline Motifs Facilitate Enamel Evolution through Matrix Subunit Compaction. doi:10.1371/journal.pbio.1000262**

